# Growth changes of tomato seedlings responding to sodium salt of α-naphthalene acetic acid and potassium salt of fulvic acid

**DOI:** 10.1038/s41598-023-31023-x

**Published:** 2023-03-10

**Authors:** Maofei Ren, Guiling Mao, Huabin Zheng, Weiqin Wang, Qiyuan Tang

**Affiliations:** 1 College of Agronomy, Hunan Agricultural University, Changsha, 410128 Hunan People’s Republic of China; 2 College of Horticulture, Shanxi Agricultural University, Taigu, 030801 Shanxi People’s Republic of China

**Keywords:** Plant sciences, Photosynthesis, Plant biotechnology, Plant stress responses

## Abstract

In present study, sodium salt of α-naphthalene acetic acid (NA), potassium salt of fulvic acid (KF) and their combinations were applied to the growth substrates of tomato seedlings (*Solanum lycopersicum* L.) under chilling stress. The changes in aboveground biomass, root attributes, pigment contents, chlorophyll fluorescence, photosynthesis, osmotic regulation substances, and antioxidant enzymes activity of the tomato seedlings in response to NA and KF were investigated. The application of NA, KF and their combination could promote the growth of plant height and stem diameter of tomato seedlings under chilling stress to varying degrees, and improve root characteristics by increasing root volume, root length and root activity, and increase dry matter accumulation. In addition, the combined use of NA and KF improved the seedling leaf chlorophyll content, qP, Fv/Fm, ΦPSII , Pn and increased the activity of antioxidant enzymes in the tomato plants. The above results suggested a synergistic effect between NA and KF to stimulate the seedlings growth and to enhance the ROS scavenging ability of tomato, which has never been reported in previous research before. However, further researches are needed to explore the physiological and molecular mechanism underlying the synergistic effect between NA and KF.

## Introduction

The population growth and economic development has aroused the demands for increasing crop productivity including field crops, fruits and vegetables^1,2^. In recent years, however, the total crop production has been continuously challenged by environmental degradation, destruction of natural ecosystems and loss of biodiversity^3–10^, and the increased production must be achieved by improving crop yield and land use efficiency. Although the genetic improvements have made great effort in increasing the crop yield potential, the yield gap in actual production, which is influenced by environmental factor, fertilizer application, and several biotic and abiotic stresses, has greatly affected the yield stability^11^. Therefore, approaches to enhance crop growth performance and to diminish the negative effects of abiotic stresses and inappropriate crop management on yield stability are desperately needed. Regulating the crop growth by plant growth regulators (PGR) is an important approach in modern agriculture^12,13^. In the past decades, several PGRs including paclobutrazol, gibberellins, chlormequat chloride and mepiquat chloride have successfully developed and adopted in the production of various crops, and their effects and regulating mechanisms on crop yield, quality and stress tolerance have been intensively studied^14–18^. The beneficial of the PGRs includes improving crop yield, modifying crop growth speed, regulating nutritional quality and enhancing stress tolerance^19–22^. But the overuse of PGRs may raise concerns of the potential health risks to consumers and environmental pollution^23^. Plant biostimulant means a material which contains substance(s) and/or microorganisms whose function when applied to plants or the rhizosphere is to stimulate natural processes to benefit nutrient uptake, nutrient efficiency, tolerance to abiotic stress, and/or crop quality, independently of its nutrient content^24^. The biostimulant obsessed a similar or better function, but with smaller risks of human health and environmental pollution as compared with PGRs, and has now widely used in the production of fruits and vegetables.

The KF is a typical biostimulant, and NA is is a synthetic auxin, known for decades and registered as a PGR, i.e., a plant protection product. KF is the active organic compound in soil humid acid. It is a highly efficient macro-molecular organic compound with short carbon chain molecular structure and high solubility^25^. KF has a low molecular weight and can be easily absorbed and utilized by crops^26^, which can not only regulate plant growth but also provide potassium for plant growth^27^. KF can promote the formation of new roots, increase the contents of chlorophyll, indolence acid and abscissa acid, promote dry matter accumulation, enhance the activity of antioxidant enzymes, reduce stomach opening, reduce transpiration rate and improve net photosynthetic rate, so as to enhance crop stress resistance and improve crop yield, quality and benefit^28^. NA is a kind of broad-spectrum, high efficiency and low toxicity plant growth regulator. NA can promote cell division and expansion, improve the rate of flowering and fruit setting, prevent falling flower and fruit^29^, expand fruit, promote early maturity, increase yield^30^, and improve quality. At the same time, NA can also effectively improve crop drought resistance, cold resistance, water logging resistance, salt and alkali resistance. Previously, the regulating effects of NA and KF have been observed in wheat^28^, soybea n^31^, cotton^32^, pepper^33^, coriander^34^, ficus religiosa^35^. Nevertheless, the combining effects ofNA and KF has remained to be unknown.

Tomato (*Solanum lycopersicum* L.) originated in Ecuador and other places in South America. The planting area and output of tomato in China ranks among the top in the world, and it is one of the common vegetables and fruits necessary for people's daily life. In the past decades, the development of industrial cultivation has greatly promoted the transition of the tomato production from open-field to greenhouse or plant factory, which largely improved the production efficiency and profits for farmers. And there are growing needs to enhance the growth of tomato seedlings via PGRs or biostimulants under intensive production condition. The present study examined the effects of NA, KF and their combinations on the seedling growth, photosynthesis and stress tolerant traits of tomato under chilling stress, we surprisingly found that the NA, KF and their combination affected the growth of tomato seedlings under chilling stress, in addition, the combined use of NA and KF significantly improved the quality of tomato seedlings. To the best of our knowledge, this is the first research that observed the synergistic effect of NA and KF on enhancing crop growth performance.

## Results

### Plant height, stem diameter, fresh weight, dry weight

**Figure 1:**
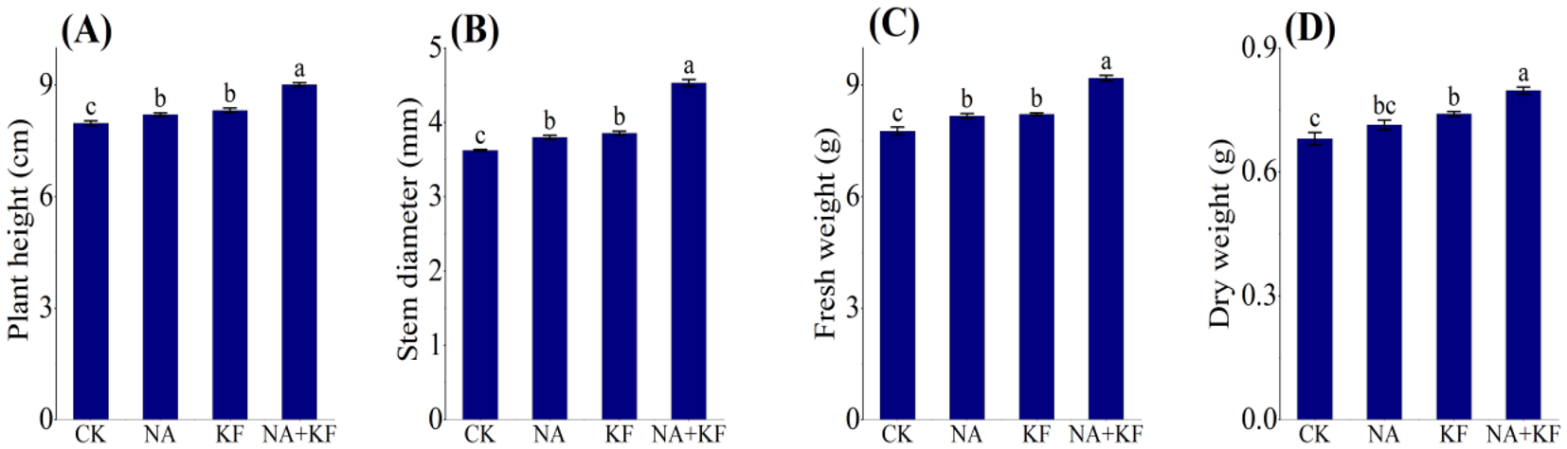
Effects of different treatments on plant height, stem diameter, fresh weight and dry weight of tomato seedlings. (**A**) Plant height, (**B**) stem diameter, (**C**) fresh weight, (**D**) dry weight, NA, 5 mg·kg^−1^ NA; KF, 60 mg·kg^−1^ KF; NA + KF, 5 mg·kg^−1^ NA and 60 mg·kg^−1^ KF. Different lowercase letters denote statistical differences among treatments of tomato seedlings at the 5% level according to LSD test. Error bars above mean indicate standard error (n = 3).

The effects of NA, KF and their combination on the aboveground seedling attributes (plant height and stem diameter), fresh weight and dry weight in seedlings of tomato was investigated. The application of NA, KF and their combination showed significant influence on the plant height, stem diameter, fresh weight and dry weight as compared with control (Fig. 1). The plant height, stem diameter, fresh weight and dry weight of tomato seedlings all had the maximum values in NA + KF treatment, and were significantly higher than other treatments. Compared with CK, an increase the plant height, stem diameter, fresh weight and dry weight of tomato seedlings for NA + KF treatment were observed by 13.04%, 25.14% 18.39% and 17.16%. In addition, the plant height, stem diameter, fresh weight and dry weight of tomato seedlings treated by the single material (NA, KF) treatment were significantly higher than CK, except for the dry weight used in NA treatment. It can be clearly deduced that NA, KF and their combination did improve plant height, stem diameter, fresh weight and dry weightin tomato seedlings, at the same time, the combined application of the NA + KF treatment was better.

### Root parameters

**Figure 2:**
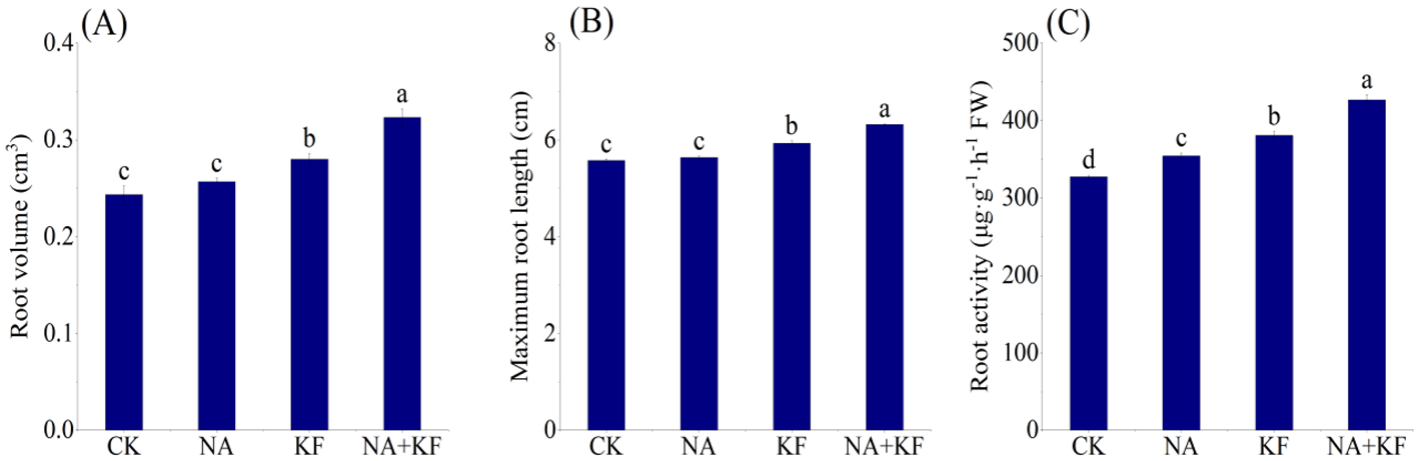
Effects of different treatments on root parameters of tomato seedlings. (**A**) Root volume, (**B**) maximum root length, (**C**) root activity, NA, 5 mg·kg^−1^ NA; KF, 60 mg·kg^−1^ KF; NA + KF, 5 mg·kg^−1^ NA and 60 mg·kg^−1^ KF. Different lowercase letters denote statistical differences among treatments of tomato seedlings at the 5% level according to LSD test. Error bars above mean indicate standard error (n = 3).

The effects of NA, KF and their combination on the seedling root parameters in seedlings of tomato was investigated. The application of NA, KF and their combination showed significant influence on the seedling root parameters as compared with control (Fig. 2). The root volume, maximum root length and root activity of tomato seedlings all had the maximum values in NA + KF treatment, and were significantly higher than other treatments. Compared with CK, an increase the root volume, maximum root length and root activity of tomato seedlings for NA + KF treatment were observed by 32.88%, 13.34% and 30.44%. In addition, the root volume, maximum root length and root activity of tomato seedlings treated by the KF treatment were significantly higher than CK. It can be clearly deduced that the combined application of the NA + KF treatment significantly improved the root volume, maximum root length and root activity in the tomato seedlings.

### Pigment contents

**Figure 3:**
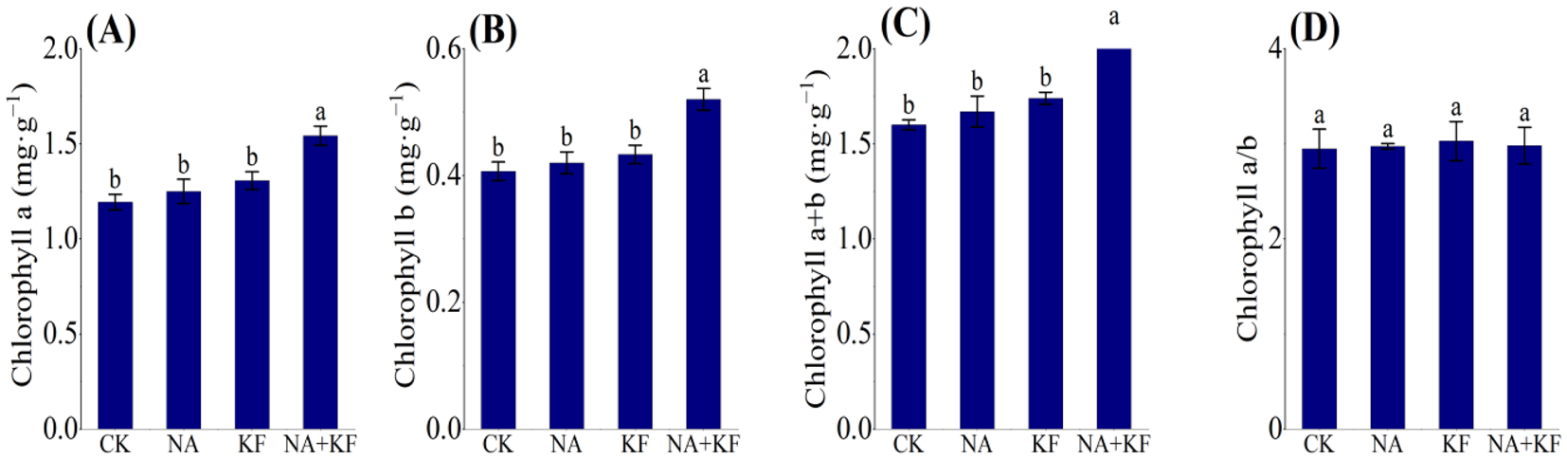
Effects of different treatments on leaf chlorophyll content of tomato seedlings. (**A**) Chlorophyll *a*, (**B**) Chlorophyll *b*, (**C**) Chlorophyll *a* + *b*, (**D**) Chlorophyll *a/b*, NA, 5 mg·kg^−1^ NA; KF, 60 mg·kg^−1^ KF; NA + KF, 5 mg·kg^−1^ NA and 60 mg·kg^−1^ KF. Different lowercase letters denote statistical differences among treatments of tomato seedlings at the 5% level according to LSD test. Error bars above mean indicate standard error (n = 3).

The effects of NA, KF and their combination on the seedling leaf chlorophyll content in seedlings of tomato was investigated. The application of NA and KF showed no significant influence on the seedling leaf chlorophyll content as compared with control (Fig. 3A–C). However, the combined application of NA and KF significantly promoted the tomato seedling leaf chlorophyll content. When compared with CK, NA + KF treatment chlorophyll a, chlorophyll b and chlorophyll *a* + *b* was raised by 29.33%, 27.86% and 28.96%. Nevertheless, the chlorophyll a/b of tomato seedling was not significant in all treatment (Fig. 3D). It can be clearly deduced that the combined application of the NA + KF treatment improved the seedling leaf chlorophyll content in the tomato plants.

### Chlorophyll fluorescence

**Figure 4:**
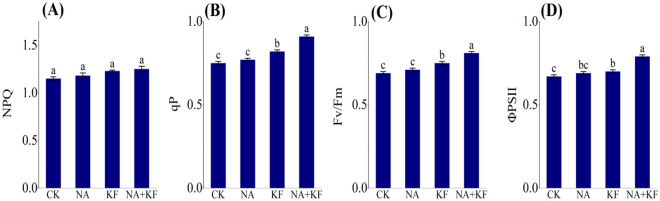
Effects of different treatments on chlorophyll fluorescence of tomato seedlings. (**A**) NPQ, (**B**) qP, (**C**) Fv/Fm, (**D**) ΦPSII, NA, 5 mg·kg^−1^ NA; KF, 60 mg·kg^−1^ KF; NA + KF, 5 mg·kg^−1^ NA and 60 mg·kg^−1^ KF. Different lowercase letters denote statistical differences among treatments of tomato seedlings at the 5% level according to LSD test. Error bars above mean indicate standard error (n = 3).

The changes in chlorophyll fluorescence of the tomato seedlings under different treatments regarding NPQ, qP, Fv/Fm and ΦPSII were shown in Fig. 4. The qP, Fv/Fm and ΦPSII of tomato seedlings all had the maximum values in NA + KF treatment, and were significantly higher than other treatments. Compared with CK, an increase the qP, Fv/Fm and ΦPSII of tomato seedlings for NA + KF treatment were observed by 21.33%, 17.39% and 17.91% (Fig. 4B–D), respectively. In addition, the qP, Fv/Fm and ΦPSII of tomato seedlings all had the second values in KF treatment, and were significantly higher than CK. Compared with CK, an increase the qP, Fv/Fm and ΦPSII of tomato seedlings for KF treatment were observed by 9.33%, 9.70% and 4.48% (Fig. 4B–D), respectively. However, The application of all treatment showed no significant influences the NPQ as compared with CK (Fig. 4A). Overall, it can be clearly deduced that the combined application of the NA + KF treatment improved the qP, Fv/Fm and ΦPSII in the tomato plants.

### Photosynthesis

**Figure 5:**
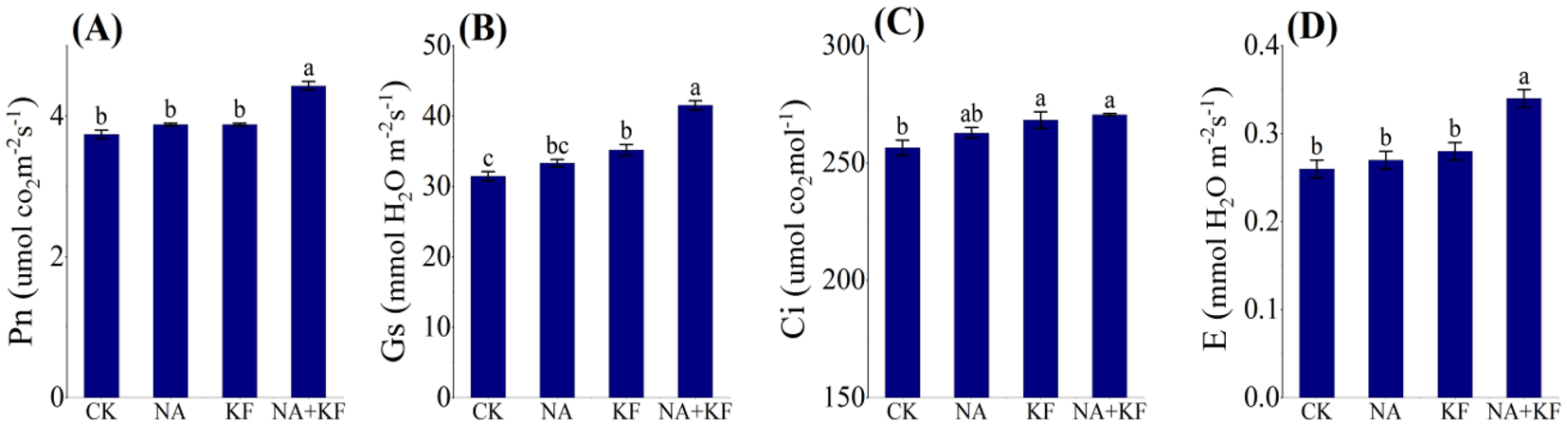
Effects of different treatments on photosynthesis of tomato seedlings. (**A**) Photosynthetic rate (Pn), (**B**) stomatal conductance (Gs), (**C**) intercellular CO_2_ concentration (Ci), (**D**) transpiration rates of leaves (**E**), NA, 5 mg·kg^−1^ NA; KF, 60 mg·kg^−1^ KF; NA + KF, 5 mg·kg^−1^ NA and 60 mg·kg^−1^ KF. Different lowercase letters denote statistical differences among treatments of tomato seedlings at the 5% level according to LSD test. Error bars above mean indicate standard error (n = 3).

The changes in photosynthesis of the tomato seedlings under different treatments regarding photosynthetic rate (Pn), stomatal conductance (Gs), intercellular CO2 concentration (Ci) and transpiration rates of leaves (E) were shown in Fig. 5. The photosynthetic parameters (Pn, Gs, Ci and E) of tomato seedlings all had the maximum values in NA + KF treatment, in addition, the photosynthetic parameters (except Ci) were significantly higher than other treatments. Compared with CK, an increase the Pn, Gs, Ci and E of tomato seedlings for NA + KF treatment were observed by 18.45%, 32.06%, 5.51% and 30.77% (Fig. 5), respectively. However, compared with CK, the Pn and E of tomato seedlings was not significantly different under single material (NA, KF) treatment (Fig. 5A,D). It can be clearly deduced that single material (NA, KF) treatment did not improve Pn in tomato seedlings, in contrary, the combined application of the NA + KF treatment significantly improved the Pn in the tomato seedlings.

### Soluble sugar, soluble protein, sucrose synthase, sucrose phosphate synthase

**Figure 6:**
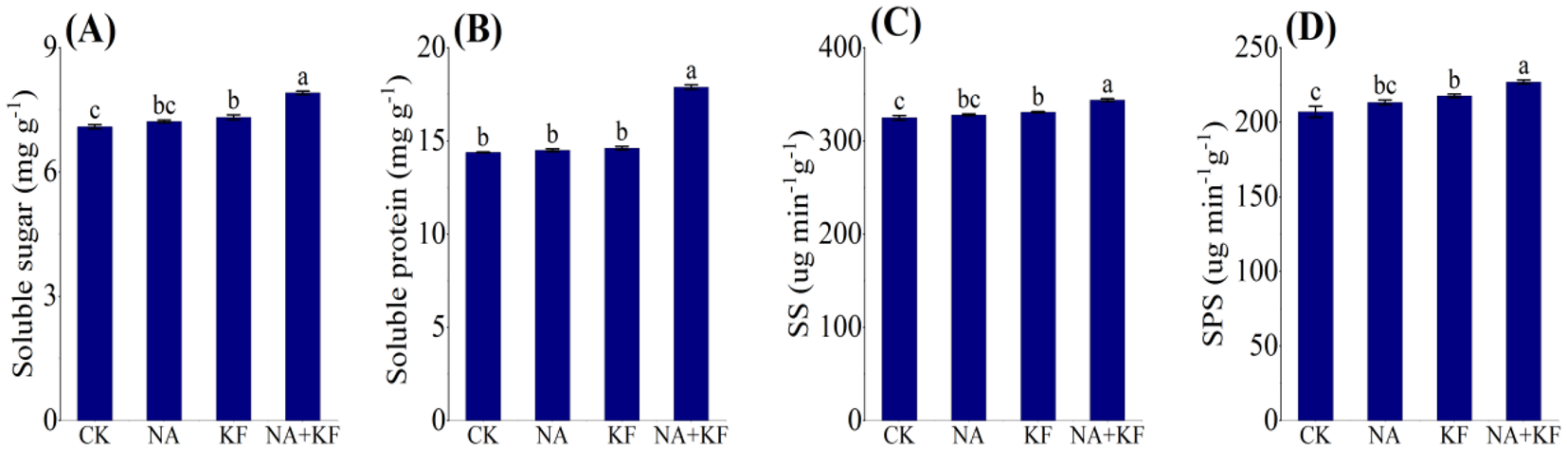
Effects of different treatments on soluble sugar, soluble protein, sucrose synthase and sucrose phosphate of tomato seedlings. (**A**) Soluble sugar, (**B**) soluble protein, (**C**) sucrose synthase (SS), (**D**) Sucrose phosphate synthase (SPS), NA, 5 mg·kg^−1^ NA; KF, 60 mg·kg^−1^ KF; NA + KF, 5 mg·kg^−1^ NA and 60 mg·kg^−1^ KF. Different lowercase letters denote statistical differences among treatments of tomato seedlings at the 5% level according to LSD test. Error bars above mean indicate standard error (n = 3).

The soluble sugar, soluble protein, sucrose synthase (SS) and sucrose phosphate synthase (SPS) of the tomato seedlings was focused on in Fig. 6, and significant variations in soluble sugar, soluble protein, SS and SPS was observed different treatments. The soluble sugar, soluble protein, SS and SPS of tomato seedlings all had the maximum values in NA + KF treatment, and were significantly higher than other treatments. Compared with CK, an increase the soluble sugar, soluble protein, SS and SPS of tomato seedlings for NA + KF treatment were observed by 11.57%, 24.34%, 5.80% and 9.67% (Fig. 8), respectively. In addition, the soluble sugar, SS and SPS of tomato seedlings had the second values in KF treatment, and were significantly higher than CK. Compared with CK, an increase the soluble sugar, SS and SPS of tomato seedlings for KF treatment were observed by 3.24%, 1.80% and 5.11% (Fig. 6A–C), respectively. It can be clearly deduced that the combined application of the NA + KF treatment improved the soluble sugar, soluble protein, SS and SPS in the tomato seedling leaves.

### Antioxidant enzyme activities and MDA content

**Figure 7:**
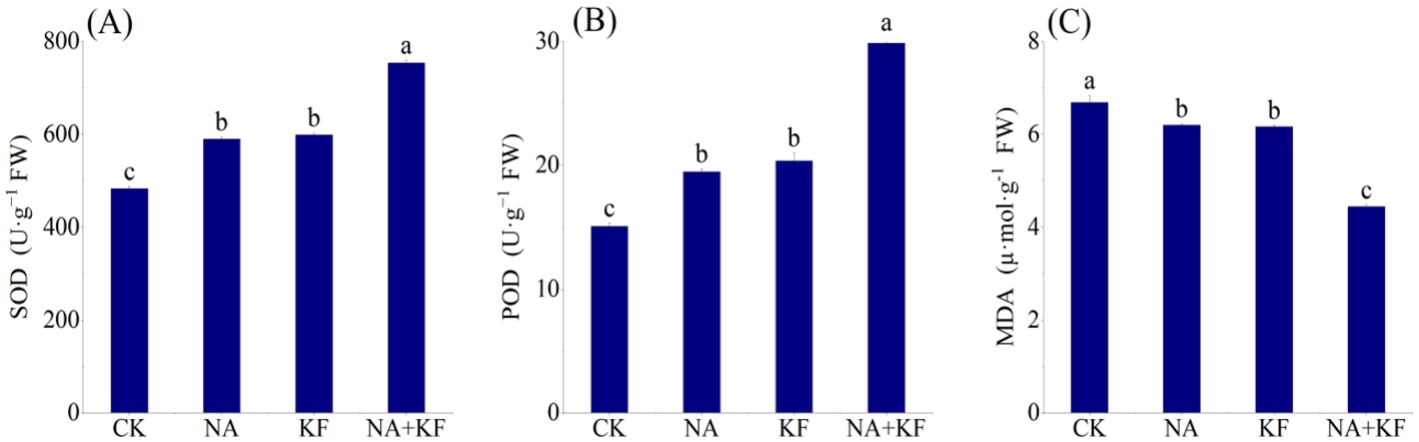
Effects of different treatments on antioxidant enzyme activities and MDA content of tomato seedlings. (**A**) Superoxide dismutase (SOD), (**B**) peroxidase (POD), (**C**) malondialdehyde (MDA), NA, 5 mg·kg^−1^ NA; KF, 60 mg·kg^−1^ KF; NA + KF, 5 mg·kg^−1^ NA and 60 mg·kg^−1^ KF. Different lowercase letters denote statistical differences among treatments of tomato seedlings at the 5% level according to LSD test. Error bars above mean indicate standard error (n = 3).

Data of the tomato seedlings antioxidant enzyme activities (SOD activity, POD activity) along with MDA content are presented in Fig. 7. The application of NA, KF and their combination showed significant influence on the seedling antioxidant enzyme activities and MDA contents as compared with control (Fig. 7). The SOD activity and POD activity of tomato seedlings all had the maximum values in NA + KF treatment, and were significantly higher than other treatments. Compared with CK, an increase the SOD activity and POD activity of tomato seedlings for NA + KF treatment were observed by 56.15% and 98.32%. In addition, the SOD activity and POD activity of tomato seedlings treated by the single material (NA, KF) treatment were significantly higher than CK. The MDA content of tomato seedlings all had the minimum values in NA + KF treatment, and were significantly lesser than other treatments. Compared with CK, an decrease the MDA content of tomato seedlings for NA + KF treatment were observed by 33.62%. In addition, the MDA content of tomato seedlings treated by the single material (NA, KF) treatment were significantly lesser than CK. It can be clearly deduced that NA, KF and their combination did improve the seedling antioxidant enzyme activities in the tomato plants, at the same time, the combined application of the NA + KF treatment was better.

### Heat map analysis

**Figure 8:**
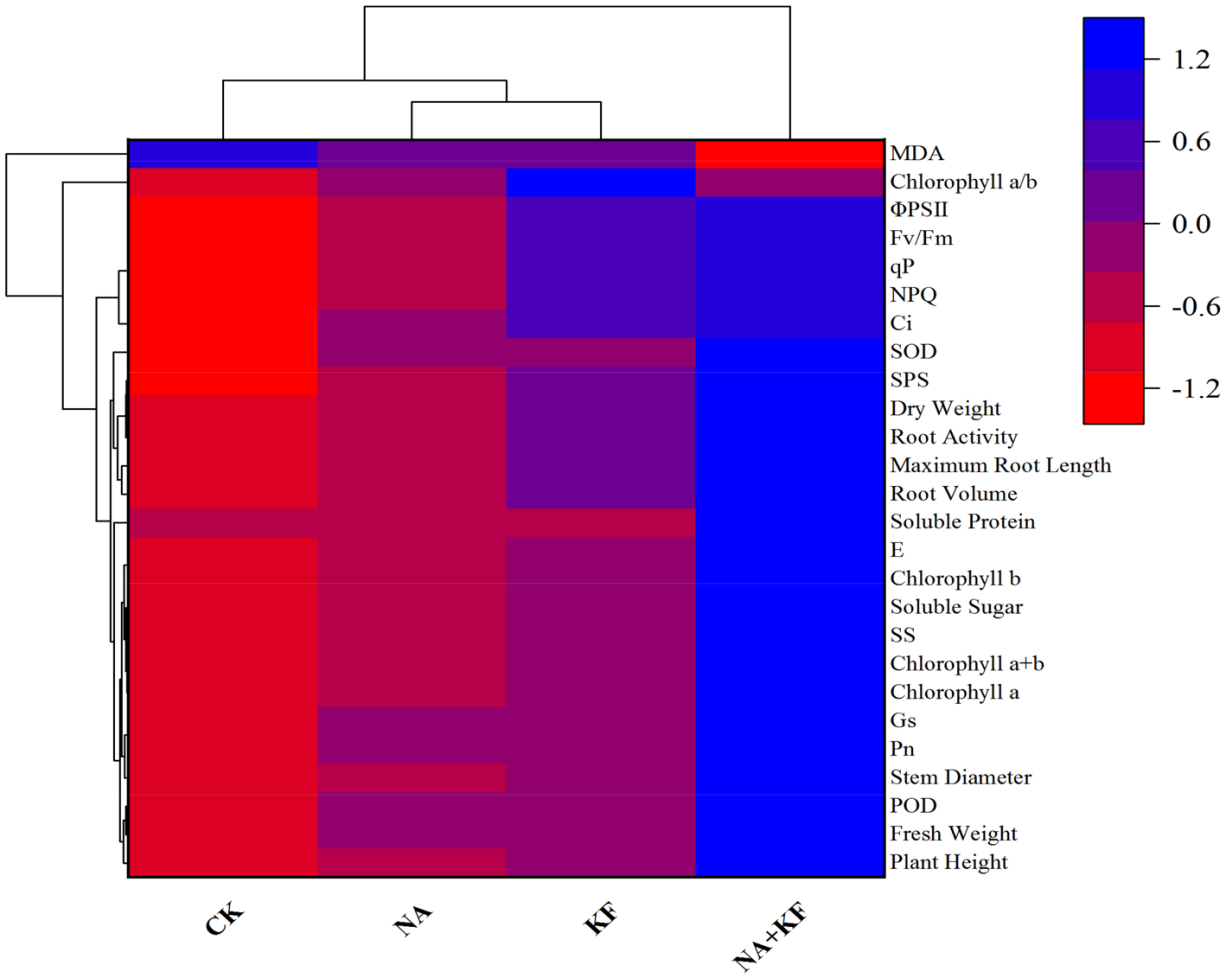
Cluster heat map analysis summarizing tomato seedlings responses to different treatments. NA, 5 mg·kg^−1^ NA; KF, 60 mg·kg^−1^ KF; NA + KF, 5 mg·kg^−1^ NA and 60 mg·kg^−1^ KF. Results are visualized using a false color scale with blue indicating an increase and red a decrease of the response parameters.

A heat map synthesizing the response of the measured parameters provided an integrated view of the effect of different treatments on the morphogenesis,antioxidant enzyme activities and photosynthetic traits of tomato seedlings (Fig. 8). In most of the measured parameters of tomato seedlings under different treatments (NA, KF and NA + KF) treatment were significantly higher than CK. In addition, in most of the measured parameters of tomato seedlings under the NA + KF treatment were significantly higher than that of the other treatments. Among the tomato seedlings, the NA and FK clusters are the closest to each other in terms of measured parameter responses, and the NA and FK clusters are equidistant from cluster CK. At the same time, cluster NA + KF is considerably separated from the other three clusters (CK, NA and FK): NA + KF reduced MDA content, and increased ΦPSII, Fv/Fm, qP, NPQ, Ci, SOD, SPS, dry Weight, root activity, maximum root length, root volume, soluble protein, E, chlorophyll b, soluble sugar, SS, chlorophyll *a* + *b*, chlorophyll *a*, Gs, Pn, stem diameter, POD, fresh weight and plant height compared to CK, NA and FK, contributing to separate the NA + KF cluster from the others.

## Discussion

### NA and KF can promote the growth of tomato seedlings under chilling stress

Chilling is one of the major abiotic stresses limiting the growth and productivity of many crops^36^. In this study, the effects of NA, KF and their combination treatments on seedling growth of tomato were investigated under chilling stress. The results of the present study were consistent with the previous researches^37^ that the plant height, stem diameter, root volume, maximum root length and root activity, fresh weight and dry weight of tomato seedling under NA and KF and their combination treatments were higher than those under CK (Figs. 1,2). NA, KF and their combination treatments can promote the growth of tomato seedlings by increasing the chlorophyll content of leaves and promoting the photosynthesis of plants under chilling stress (Figs. 3,5).

Under low temperature conditions, the root strength of tomato seedlings directly determines the growth status of the plants^38^. The results of the NA and KF and their combination treatments can result in development of the root system, expansion and elongation of the root and improve root vitality leading to improved uptake of water and nutrients^39^. Apart from that, the phenomenon in this may have some relationship to the abundance of organic carbon and potassium elements in KF, and KF application increases the nutrients, availability^26^, and it allows nutrients to interact with each other and divides them into the simplest forms attached by the KF electrolyte. However, the physiological mechanism of how NA and KF and their combination treatments affect soil physicochemical properties and then plant root growth and development remains to be further studied.

### NA and KF can improve the chilling tolerance of tomato seedlings

Soluble sugar and soluble protein are the important osmotic regulation substances in plants because of its strong water absorption^40^, which can reduce the freezing point of cell liquid and reduce the water loss ability of protoplasm, could directly or indirectly regulate the growth and development process of plants^41^. Therefore, soluble sugar and soluble protein play an important role in plant resistance to chilling stress^42^. This research found that the combined application of the NA + KF treatment improved the soluble sugar and soluble protein in the tomato seedling leaves (Fig. 6A,B). In addition, we found that the combined use of NA and KF improved SS and SPS (Fig. 6C,D), and thus beneficial to the synthesis and accumulation of soluble sugar.

Under chilling stress, plants can accelerate the dissipation process of excessive excitation energy through self-regulation^43^. However, due to the limitation of self-regulation, there is still a certain proportion of excitation energy that cannot be effectively utilized or dissipated, and part of this energy will be converted into electric energy, leading to excessive production of reactive oxygen species (ROS). The excess ROS will react with the plasma membrane to form MDA, destroy the plant membrane system, cause the deformation of chloroplasts and other organs, lead to protein degeneration, DNA damage, enzyme passivation, and disrupt various physiological processes such as Calvin cycle. It has been shown that NA and KFcan effectively alleviate the ROS generation caused by stress such as salt stress, drought, heavy metal and senescence^44–46^. This research found that NA, KF and their combination did improve the seedling antioxidant enzyme activities and decreased MDA accumulation in the tomato plants under chilling stress. Nevertheless, further study is needed to explore the physio-biochemical and molecular mechanisms of NA, KF and their combination treatments in crop production in enhancing chilling resistance.

### The synergistic effect of NA and KF on tomato seedlings

In this study, the single application of NA and KF showed positive effects on the various indicators of tomato seedlings as compared with control. Nevertheless, the innovative findings of this study, the combined application of NA and KF showed significant positive effects on the fresh weight, dry weight, root parameters, chilling tolerance of tomato seedlings plants cultivated under chilling stress. In other words, the synergistic effects of NA and KF on the seedling growth performance of tomato under chilling stress, and the mechanism behind this synergy mechanism has not been studied before. The growth of plants is inseparable from its good photosynthesis. Photosynthetic pigment is the material basis of photosynthesis in plants^47^, and its content directly determines the ability of plants to absorb and fix light energy, which is the direct expression of the strength of plant photosynthetic capacity^48^. The results of the application results of the single application of NA and KF increased the chlorophyll content of seedling leaves, but had no significant effect, compared with the control (Fig. 3). However, what's interesting is that the combined application of NA and KF significantly promoted the tomato seedling leaf chlorophyll content, and it also indicated that NA and KF had a synergistic effect on promoting chlorophyll formation in plants under chilling stress^49^. The combined application of NA and KF played an important role in maintaining the integrity of chloroplast structure and function under chilling stress.

The chlorophyll content of plant leaves directly affects its photosynthetic fluorescence parameters^50^. Photosynthetic fluorescence parameters are an effective way to study the photosynthetic physiological status of plants and have been widely used in monitoring the photosynthetic performance of various plants^51–53^. qP reflects the proportion of light energy absorbed by ΦPSII for photochemical reaction, while NPQ reflects the proportion of light energy absorbed by ΦPSII for heat dissipation^54,55^. Chilling stress resulted in the decrease of qP and the increase of NPQ in plant leaves^56^. However, the combined use of NA and KF alleviated the decrease of qP, but further promoted the increase of NPQ (Fig. 4A,B). The results indicated that NA and KF was beneficial to maintain the photoconversion of ΦPSII, promoted the dissipation of excessive excitation energy, and alleviated the pressure of excessive excitation energy on the photosynthetic system. In this study, the combined use of NA and KF in tomato seedlings under chilling stress increased Fv/Fm and ΦPSII (Fig. 4C,D), and then promoted Pn (Fig. 5A)^57^. However, the improvement of photosynthetic pigment and chlorophyll fluorescence parameters by the combination of NA and KF is caused by the complex physiological mechanism of plant photosynthesis, rather than the simple additive effect, which still needs to be further studied.

## Conclusions

The application of NA and KF and their combination could promote the growth of plant height and stem diameter of tomato seedlings under chilling stress to varying degrees, and improve root characteristics by increasing root volume, root length and root activity, and increase dry matter accumulation. In addition, the combined use of NA and KF improved the seedling leaf chlorophyll content, qP, Fv/Fm, ΦPSII , Pn and increased the activity of antioxidant enzymes in the tomato plants. The above results suggested a synergistic effect between NA and KF to stimulate the growth of tomato seedlings under chilling stress, which has never been reported in previous research before. The present study provided new avenues for NA and KF in crop production. Nevertheless, further study are needed to explore the physio-biochemical and molecular mechanisms of NA, KF and their combination treatments in crop production.

## Materials and methods

### Plant material

The seeds of an elite tomato variety ‘Jinfen’ were obtained from Henan Yuyi Seed Industry Science and Technology Development Co., Ltd.

### Experimental design

The tomato seeds were soaked in distilled water with the water temperature of 55 °C for 18 h and then germinated on two layers of filter papers in a germination box at 28 °C in a growth chamber until radicle protrusion. The germinated seeds were then sown on 50-cell tray (length/width: 54 cm/28 cm) with nutritional soil (peat:vermiculite:perlite = 3:2:2). When the first leaf of tomato seedlings were fully expanded, the seedling trays were transferred to an artificial climate chamber (YHMR-1000, Ningbo Yanghui Instrument Co., Ltd, China) with 12 h photoperiod (200 μmol m^−2^ s^−1^), 25 °C/18 °C (daytime/night), and 70%/80% (daytime/night) relative humidity.

The experiments were arranged in complete-randomized design with three replications, and 30 plants for each replication. At three-leaf stage, the tomato seedlings with similar growth performance were selected and transplanted into the nutrient bowl (Bottom diameter/calibre/height:8 cm/10 cm/10 cm) with nutritional soil (peat:vermiculite:perlite = 3:2:2), and further treatments with 12 h photoperiod (200 μmol m^−2^ s^−1^), 12 °C/6 °C (daytime/night), and 70%/80% (daytime/night) relative humidity.

The treatments includes NA (5 mg·kg^−1^ NA), KF (60 mg·kg^−1^ KF), NA + (5 mg·kg^−1^ NA and 60 mg·kg^−1^ KF), the distilled water were used as control. At 1, 5, 10, 15, and 20 days after transplanting, 30 ml of the treatment solution were added, and the concentration of each treatment solution was optimized in a preliminary experiment. The detailed information of the treatments was shown in Table 1.

**Table 1:** Experimental design and treatment combination of α-NaNAA and KF.

Treatment	α-NaNAA (mg·kg^−1^)	KF (mg·kg^−1^)
CK	0	0
NA	5	0
KF	0	60
NA + KF	5	60

The purity of KF (Xinjiang Shuanglong Humic Acid Factory, Wulumuqi, Xinjiang, China.) was 45%. The purity of α-NaNAA (Henan Zhenrui Biological Technology Co., Ltd, Zhoukou, Henan, China) was 99.99%, NA, 5 mg·kg^−1^ α-NaNAA; KF, 60 mg·kg^−1^ KF; NA + KF, 5 mg·kg^−1^ α-NaNAA and 60 mg·kg^−1^ KF.

After 21 days of transplanting, the tomato seedlings plants under different treatments were sampled, and the related indexes were determined. The sample parts were immediately frozen in liquid nitrogen and stored at −80 °C until analysis.

### Observations

#### Plant growth parameters

Plant height, stem diameter, root length, root volume, fresh weight, and dry weight. Plants with consistent growth were selected for sampling, and ten plants were selected for each replicate.. The measurement determination was done according to the method described by Zheng et al.^58,59^.

#### Root activity

Using TTC REDOX method^60^, 0.5 g of root tip was taken and immersed in a solution of 0.4% TTC and 5 mL phosphoric acid buffer (pH 7.0), and kept dark for 2 h at 37 °C. After that, 1 mol·L^−1^ sulfuric acid was added to stop the reaction. Put the root into a mortar, add ethyl acetate 3–4 mL to fully grind, move the red extract into the calibration tube, rinse with ethyl acetate several times and constant volume to 10 mL, use phototypesetter to compare colors at wavelength 485 nm, use blank test (the root was treated with sulfuric acid first) as reference to measure the absorbance, check the standard curve, TTC reduction amount can be calculated.

#### Chlorophyll assay

The leaf samples were mixed with sodium phosphate buffer (50 mM, pH 6.8) and ground in an ice bath. The superannuate was mixed with 95% ethanol and kept in the dark for 30 min before being centrifuged at 1000×*g* under 4 °C for 15 min. The absorbance at wavelengths 665 nm and 649 nm was measured using a phototypesetter (Hitachi, U-2900)^61^.

### Chlorophyll fluorescence

Chlorophyll fluorescence was measured by a FluorPen 110/D (PSI, Photon Systems Instruments, Czech Republic). At 21 days after treatment, the chlorophyll fluorescence detection method was according to Oxborough^62^. In brief, the plants were dark adapted for 20 min prior determination of minimum (F0) and maximum (Fm) fluorescence.Then, leaves wereadapted to PPFD of 500 mmol m^−2^ s^−1^ and a saturating pulse of 0.8 s with > 6000 mmol m^−2^ s^−1^ was applied in order to determine the minimum (F'0), maximum (F'm) and the steady-state (Fs) fluorescence in light adapted conditions. Each treatment was repeated three times.The non-photochemical quenching (NPQ) due to dissipation of excess light energy was calculated as Eq. (1). The coefficient of photochemical quenching (qP) due to an estimate of open PSII reaction centers was calculated as Eq. (2). The maximum quantum yield of PSII photochemistry (Fv/Fm) was determined as Eq. (3). The quantum yield of PSII (ΦPSII) was calculated according to Eq. (4).

1 $$ {\text{NPQ }} = {\text{ Fm }}/{\text{F}}^{\prime}{\text{m}}-{1} $$

2 $$  {\text{qP }} = \, ({\text{F}}^{\prime}{\text{m}}- {\text{Fs}}) \, /{\text{ F}}^{\prime}{\text{m}}- {\text{F}}^{\prime}0 $$

3 $$ {\text{Fv}}/{\text{Fm }} = { 1 } - {\text{ F}}0/{\text{Fm}} $$

4 $$  \Phi {\text{PSII }} = \, ({\text{F}}^{\prime}{\text{m}}-  {\text{Fs}}) \, /{\text{F}}^{\prime}{\text{m}} $$

### Photosynthetic parameters analysis

At 21 days after treatment, the second fully expanded leaf was used for determination of photosynthetic rate (Pn), stomatal conductance (Gs), intercellular CO_2_ concentration (Ci) and transpiration rates of leaves (E) using an infrared gas analyzer (LI-6400, Li-COR, Lincoln, OR, USA) in a growth chamber with a constant temperature of 25 °C, 400 mg·kg^−1^ CO_2_ concentration and 70% relative humidity^63^.

#### Soluble sugar, soluble protein

soluble sugar was measured by the Song^62^ method. Samples (0.1 g fresh leaves) were put into a test tube, to which 10 mL of distilled water was added and mixed. After 30 min in a water bath at 95 °C, the supernatant was collected. This step was repeated three times, and then distilled water was added to a volume of 10 mL. The soluble sugar content was determined with the sulfuric acid anthrone method at a wavelength of 620 nm.

Soluble protein was measured by the Song^62^ method. Samples (0.1 g fresh leaves) were ground up in a mortar with liquid nitrogen, to which 6 mL of a phosphatebuffered solution (pH 7.0) was added. The extract was centrifuged at 15,000×*g* for 20 min at 4 °C, and 0.2 mL of the supernatant was combined with 9.8 mL of a Coomassie brilliant blue G-250 solution (0.1 g·L^−1^). After 5 min, the soluble protein content was determined at a wavelength of 595 nm.

#### Sucrose synthase (SS), sucrose phosphate synthase (SPS)

Sucrose synthase (SS) and sucrose phosphate synthase (SPS) was extracted using assay kits (ZC-S0507, ZC-S0508, Shanghai ZCIBIO Technology Co., Ltd., Shanghai, China), and the activity was measured at 480 nm by the ultraviolet spectrophotometer. The catalytic production of 1 μg sucrose per g tissue per minute is defined as a unit of enzyme activity.

#### Superoxide dismutase (SOD) assay

The leaf samples were mixed with three sodium phosphate buffer (50, pH 7.4) and ground in an ice bath before being centrifuged at 15,000×*g* under 4 °C for 30 min. The superannuate was mixed with methamphetamine–methamphetamine buffer (100 mM, pH 7.4), EDTA/MnCl_2_ (100 mM/50 mM, pH 7.4), 2-mercaptoethanol (10 mM), and NADH (7.5 mM). The SOD activity was determined by a phototypesetter (Hitachi, U-2900) at 340 nm. In this study, one unit of SOD was defined as the enzyme activity that inhibits 50% of the NADH oxidation rate in blank samples^64^.

#### Peroxidase (POD) assay

The leaf samples were mixed with sodium phosphate buffer (50 mM, pH 6.8, containing 1 mM hydrodynamic) and ground in an ice bath. The ground sample was centrifuged at 6000×*g* under 4 °C for 25 min. The superannuate was mixed with titanium chloride (0.1% v/v dissolved in 20% (v/v) H_2_SO_4_) and centrifuged at 4000×*g* at room temperature for 30 min. The POD activity was determined by a phototypesetter (Hitachi, U-2900) at 410 nm^65^.

#### Malondialdehyde (MDA) assay

The leaf samples were ground in trigonometrical acid (TCA, 5% w/v) before being centrifuged at 10,000×*g* under 20 °C for 5 min. The superannuate was mixed with barbiturate acid (0.5% w/v, containing 20% w/v TCA) and placed in a water bath at 95 °C for 30 min before centrifuging at 3000×*g* under room temperature for 10 min. The MDA activity was determined by a spectrophotometer (Hitachi, U-2900) at 532 and 600 nm^66,67^.

### Statistical analysis

A one-way analysis of variance was conducted to test the effects of NA and KF on Tomato Seedling using IBM SPSS Statistics 20 (IBM, Inc., Chicago, IL, USA). test was used to make post-choc multiple comparisons at α = 0.05. Different alphabetical letters are used in figures and tables for showing significant differences.

### Ethics statement

We ensure that we have permission to collect tomato plants, and experimental research and field studies on plants including the collection of plant material, comply with relevant institutional, national, and international guidelines and legislation (Supplementary Information).

## Supplementary Information

Supplementary Information.

## Data Availability

All data generated or analysed during this study are included in this published article (and its supplementary information files). We ensure that we have permission to collect tomato plants, and experimental research and field studies on plants including the collection of plant material, comply with relevant institutional, national, and international guidelines and legislation.
